# The 287,403 bp Mitochondrial Genome of Ectomycorrhizal Fungus *Tuber calosporum* Reveals Intron Expansion, tRNA Loss, and Gene Rearrangement

**DOI:** 10.3389/fmicb.2020.591453

**Published:** 2020-12-09

**Authors:** Xiaolin Li, Lijiao Li, Zhijie Bao, Wenying Tu, Xiaohui He, Bo Zhang, Lei Ye, Xu Wang, Qiang Li

**Affiliations:** ^1^Soil and Fertilizer Institute, Sichuan Academy of Agricultural Sciences, Chengdu, China; ^2^School of Food and Biological Engineering, Chengdu University, Chengdu, China; ^3^College of Life Sciences, Henan Agricultural University, Zhengzhou, China

**Keywords:** *Tuber*, mitochondria, phylogenetic analysis, gene rearrangements, evolution 3

## Abstract

In the present study, the mitogenome of *Tuber calosporum* was assembled and analyzed. The mitogenome of *T. calosporum* comprises 15 conserved protein-coding genes, two rRNA genes, and 14 tRNAs, with a total size of 287,403 bp. Fifty-eight introns with 170 intronic open reading frames were detected in the *T. calosporum* mitogenome. The intronic region occupied 69.41% of the *T. calosporum* mitogenome, which contributed to the *T. calosporum* mitogenome significantly expand relative to most fungal species. Comparative mitogenomic analysis revealed large-scale gene rearrangements occurred in the mitogenome of *T. calosporum*, involving gene relocations and position exchanges. The mitogenome of *T. calosporum* was found to have lost several tRNA genes encoding for cysteine, aspartate, histidine, etc. In addition, a pair of fragments with a total length of 32.91 kb in both the nuclear and mitochondrial genomes of *T. calosporum* was detected, indicating possible gene transfer events. A total of 12.83% intragenomic duplications were detected in the *T. calosporum* mitogenome. Phylogenetic analysis based on mitochondrial gene datasets obtained well-supported tree topologies, indicating that mitochondrial genes could be reliable molecular markers for phylogenetic analyses of Ascomycota. This study served as the first report on mitogenome in the family Tuberaceae, thereby laying the groundwork for our understanding of the evolution, phylogeny, and population genetics of these important ectomycorrhizal fungi.

## Introduction

The *Tuber* genus is a diversified lineage of truffle-forming fungi that produce hypogeous fruiting bodies. Truffles are regarded as prized food delicacies because of their unique flavors (Zhang et al., 2016; Caboni et al., 2020). As ectomycorrhizal fungus, *Tuber* species must form ectomycorrhiza with their host plants to complete their life cycles. It was reported that symbiosis with truffles and other ectomycorrhizal fungi could promote the growth of host plants and enhance the tolerance of host plants to pathogenic and abiotic stresses (Luo et al., 2009; Sebastiana et al., 2018; Zhang et al., 2019; Li X. et al., 2020). In return, plants provide carbon sources for truffles to grow and reproduce. The formation of this symbiotic relationship in nature plays an important role in maintaining the balance of forest ecosystem and promoting the carbon natural cycle (Franco et al., 2014; Corrales et al., 2018). Truffle, expecially *T. melanosporum*, has been used as model species to study the evolution, genetics, and ecological adaptation of ectomycorrhizal fungi (Martin et al., 2010; Murat et al., 2018b; Zarivi et al., 2018). Genome analyses revealed peculiar features of truffles, such as heterothallism, few genes coding lignocellulose-degrading enzymes, were closely related to the ectomycorrhizal life patterns of truffles (Martin et al., 2010; Rubini et al., 2011; Murat et al., 2018a, b). However, the mitochondrial gene characteristics of truffles are still unknown, which limits our comprehensive understanding of the genetic information and evolution of truffles. *Tuber calosporum* was found in southwest China, which was described by Wan et al. (2016). *Tuber calosporum* lives in soil under mixed forest with *Pinus yunnanensis* as dominant species. Phylogenetic analysis found that the *T. calosporum* belonged to the Macrosporum group (Wan et al., 2016).

As additional genetic component of eukaryotes cells, the mitochondrial genome (mitogenome) has been reported playing an important regulatory role in the process of stress resistance, growth and development, aging, and death (Ding et al., 2019; Luevano-Martinez et al., 2019). In addition, mitogenome features, such as the several available molecular markers, uniparental inheritance, have promoted the mitogenome becoming a powerful tool for studying the phylogeny and evolution of eukaryotic species (Andersen and Balding, 2018; Fourie et al., 2018; Li et al., 2018b, 2020a). The repetitive sequence, intron information, tRNA structure, and gene arrangement of the mitogenome also provide useful information for understanding the evolution of species (du Toit et al., 2017; Li et al., 2018a, 2020b). However, compared to the available mitogenomes of animals (>9,000 mitogenomes in database), the available mitogenome of fungi (<700 mitogenomes in database) is far from enough and is even less than the studied nuclear genomes of fungi (>5,000 genomes in database). So far, only two complete mitogenomes from Pezizales have been published in the NCBI database, including *Pyronema omphalodes* (Nowrousian, 2016) and *Morchella importuna* (Liu et al., 2019). No mitogenome of Tuberaceae has been reported. The rapid development of the next-generation sequencing (NGS) technology and the third-generation sequencing technology (Giordano et al., 2017; Zascavage et al., 2019) provides us with the possibility to obtain complex fungal mitogenomes, which promotes our understanding of fungal evolution and phylogeny.

In the present study, the complete mitogenome of *Tuber calosporum* was sequenced using the NGS technology and successfully assembled. The aims of this study are (1) to reveal the features of *T. calosporum* mitogenomes and the similarities or variations between Pezizales mitogenomes; and (2) to analyze the phylogenetic status of *Tuber calosporum* in *Ascomycota* based on a combined mitochondrial gene set. As the first reported mitogenome in the Tuberaceae, the mitogenome of *T. calosporum* will help to understand the phylogeny and evolution of truffles and provide reference for the acquisition of more truffle mitogenomes.

## Materials and Methods

### Assembly and Annotations of Mitogenome

The raw sequencing data of *T. calosporum* were obtained from the 90 mushroom genome sequencing project (Li H. et al., 2018), under the following Sequence Read Archive (SRA) accession numbers: SRR5804115 and SRR5804116. The raw sequencing data of *T. calosporum* was generated by the Illumina HiSeq 4000 platform, and a total of 22.5 Gbp data were obtained. A series of quality control steps were conducted to obtain clean reads from the raw sequencing data, including removal of adapters (Schubert et al., 2016) and filtering sequences with low quality value. The obtained clean reads were used to assemble the complete mitogenome of *T. calosporum* by SPAdes 3.9.0 (Bankevich et al., 2012). The software MITObim V1.9 (Hahn et al., 2013) was used to fill in the gaps between the contigs obtained in the previous step. Since organelle sequences usually have more copies than nuclear gene sequences, the coverage is generally higher than that of nuclear genome sequences when assembled. In addition, we also used MIRA and NOVO Plasty to test the assembly of this study. All the software obtained mitochondrial sequences identical to this study, which proves that the mitogenome obtained in the present study is reliable. The obtained complete mitogenome of *T. calosporum* was further annotated according to methods we previously described (Li et al., 2018a). Briefly, the protein-coding genes (PCGs), introns, rRNA genes, and tRNA genes of the *T. calosporum* mitogenome were initially annotated using the MITOS (Bernt et al., 2013) and MFannot (Valach et al., 2014), using the Mold, Protozoan, and Coelenterate Mitochondrial Code (genetic code 4). Then the PCGs were predicted or modified by using the NCBI Open Reading Frame Finder (Coordinators, 2017) and further annotated by BLASTP searches against the NCBI non-redundant protein sequence database (Bleasby and Wootton, 1990). Intron-exon borders of PCGs were verified using exonerate v2.2 (Slater and Birney, 2005). The tRNA genes in the *T. calosporum* mitogenome were also predicted by the tRNAscan-SE v1.3.1 (Lowe and Chan, 2016). The OGDraw v1.2 software (Lohse et al., 2013) was used to draw graphical maps of the *T. calosporum* mitogenome.

### Sequence and Repetitive Elements Analyses of the *T. calosporum* Mitogenome

The base composition of the *T. calosporum* mitogenome was calculated using DNASTAR Lasergene v7.1^1^. Strand asymmetries of the *T. calosporum* mitogenome and other related mitogenomes were assessed according to the following formulas: AT skew = [A – T] / [A + T] and GC skew = [G – C] / [G + C] (Li et al., 2019a). To identify whether there were interspersed repeats or intragenomic duplications of large fragments throughout the *T. calosporum* mitogenome, we conducted BLASTN searches (Chen et al., 2015) of the mitogenome against itself based on an *E*-value of <10^–10^. In addition, Tandem Repeats Finder (Benson, 1999) was used to detect tandem repeats (>10 bp in length) in the *T. calosporum* mitogenome. REPuter (Kurtz et al., 2001) was used to identify forward (direct), reverse, complemented, and palindromic (reverse complement) repeats in the *T. calosporum* mitogenome. We also conducted BLASTN searches of the *T. calosporum* mitogenome against its published nuclear genome (QFET00000000.1) (Li H. et al., 2018) to identify if there were gene segments naturally transferring between nuclear and mitochondrial genomes.

### Comparative Mitogenome and Phylogenetic Analyses

The genome sizes, base composition, gene numbers, intron numbers, gene content between different Pezizales mitogenomes, and the largest mitogenome in Basidiomycota (*Rhizoctonia solani*) were compared to assess variations or conservativeness of mitogenomes. To investigate the phylogenetic status of *T. calosporum* in the Ascomycota phylum, we constructed a phylogenetic tree of 104 species based on the combined mitochondrial gene set (15 core PCGs + two rRNA genes) (Li et al., 2018d). *Rhizoctonia solani* (Losada et al., 2014) and *Blastosporella zonata* (Nieuwenhuis et al., 2019) from the Basidiomycota phylum and *Chytriomyces confervae* (van de Vossenberg et al., 2018) from Chytridiomycota were set as outgroups. MAFFT v7.037 software (Katoh et al., 2019) was used to align individual mitochondrial genes. Then the aligned mitochondrial genes were concatenated into a combined mitochondrial gene set using SequenceMatrix v1.7.8 (Vaidya et al., 2011). A partition homogeneity test was used to detect potential phylogenetic conflicts among different mitochondrial genes. PartitionFinder 2.1.1 (Lanfear et al., 2017) was used to determine best-fit models of evolution and partitioning schemes for the mitochondrial gene set. Bayesian inference (BI) and maximum likelihood (ML) methods were used to construct phylogenetic trees. BI analysis was conducted using the MrBayes v3.2.6 (Ronquist et al., 2012) software and ML analysis was performed with RAxML v 8.0.0 (Stamatakis, 2014). When we conducted BI analysis, two independent runs with four chains (three heated and one cold) each were conducted simultaneously for 2 × 10^6^ generations. Each run was sampled every 100 generations. We assumed that stationarity had been reached when the estimated sample size (ESS) was greater than 100, and the potential scale reduction factor (PSRF) approached 1.0. The first 25% samples were discarded as burn-in, and the remaining trees were used to calculate Bayesian posterior probabilities (BPP) in a 50% majority-rule consensus tree. Bootstrap values (BS) were assessed through an ultrafast bootstrap approach with 10,000 replicates.

### Data Availability

The complete mitogenome of *T. calosporum* was deposited in the GenBank database under the accession number MT028548.

## Results

### Mitogenome Features and Composition

**FIGURE 1:**
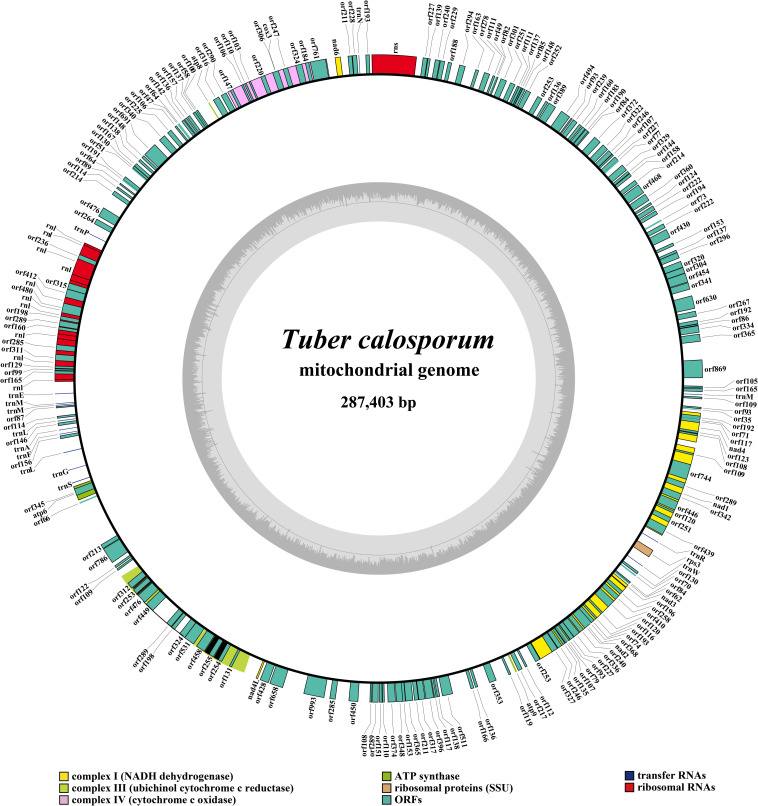
Circular map of the mitochondrial genome of *Tuber calosporum*. Genes are represented by different colored blocks.

**FIGURE 2:**
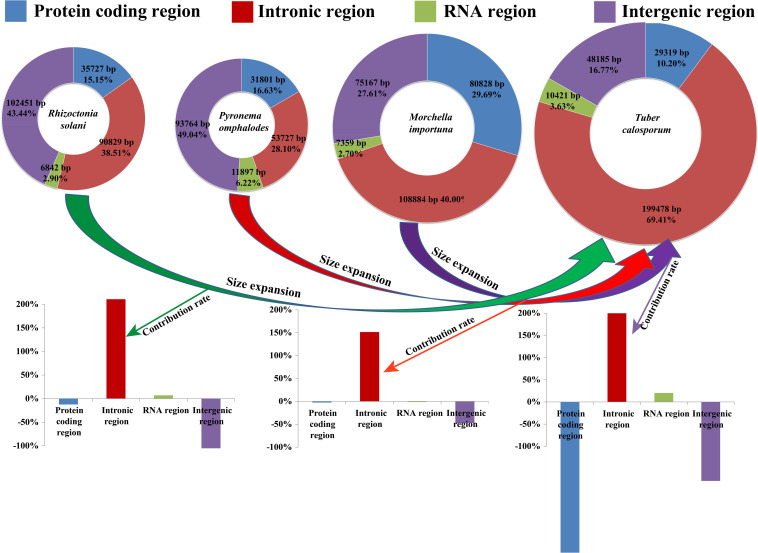
The protein-coding, intronic, intergenic, and RNA gene region proportions of the entire mitochondrial genomes of *Tuber calosporum*, *Pyronema omphalodes*, *Morchella importuna*, and *Rhizoctonia solani*. The bottom panel shows the contribution of different gene regions to the size expansion of the *T. calosporum* mitogenome.

The complete mitogenome of *T. calosporum* was composed of circular DNA molecules with a size of 287,403 bp (Figure 1). The GC content was 29.92%. Both the AT skew and GC skew were positive in the *T. calosporum* mitogenome (Table 1). We detected two pairs of overlapping ORFs in the mitogenome of *T. calosporum*, one of which located across the neighboring genes *orf228* and *orf211* (−10 bp) and the other of which was located between *orf62* and *orf84* (−28 bp) (Supplementary Table 1). The length of the intergenic sequences ranged from 0 to 6,501 bp, and the longest intergenic sequence was located across the neighboring genes orf66 and *orf213* gene. Intronic regions occupied the largest proportion of the *T. calosporum* mitogenome, reaching 69.41% (Figure 2). Intergenic region was the second largest region, accounting for 16.76%. The protein coding region accounted for 10.20% of the entire mitogenome. The RNA genes (including tRNAs and rRNAs) were 10,421 bp long in total, accounting for 3.63% of the whole mitogenome. Comparative mitogenomic analysis indicated that intron gain was the primary factor that contributed to the size expansion of the *T. calosporum* mitogenome. Compared with the other three mitogenomes, the protein coding region and intergenic region reduced in *T. calosporum.*

**TABLE 1:** Characteristics of 4 mitogenomes from Ascomycota and Basidiomycota.

Item	*Tuber calosporum*	*Pyronema omphalodes*	*Morchella importuna*	*Rhizoctonia solani*
Phylum	Ascomycota	Ascomycota	Ascomycota	Basidiomycota
Order	Pezizales	Pezizales	Pezizales	Cantharellales
Family	Tuberaceae	Pyronemataceae	Morchellaceae	Ceratobasidiaceae
Accession number	MT028548	KU707476	MK527108	KC352446
Genome size (bp)	287,403	191,189	272,238	235,849
GC content (%)	29.92	42.98	39.95	35.91
AT skew	0.023	0.012	0.020	−0.004
GC skew	0.077	0.044	0.042	−0.002
No. of PCGs	43	29	126	88
No. of introns	58	21	34	31
Intronic ORFs	170	19	39	39
No. of rRNAs	2	2	2	2
No. of tRNAs	14	26	31	26

### Protein Coding Genes, tRNAs, rRNAs, and Codon Analysis

A total of 43 free standing (non-intronic) protein coding genes (PCGs) were detected in the mitogenome of *T. calosporum*, including 14 core PCGs for energy metabolism, one *rps3* gene for transcriptional regulation, eight PCGs containing the LAGLIDADG homing endonuclease domain, 10 PCGs with the GIY-YIG homing endonuclease domain, three genes encoding DNA-directed RNA polymerase, and seven PCGs with unknown functions (Supplementary Table 1). A total of 58 introns were detected in the *T. calosporum* mitogenome, which were distributed in *atp6*, *cob*, *cox1*, *cox2*, *cox3*, *nad1*, *nad2*, *nad4*, *nad5*, and *rnl* genes. One hundred and seventy intronic open reading frames (ORFs) were detected in these introns, including 110 intronic ORFs containing LAGLIDADG homing endonuclease domain, 56 ORFs containing GIY-YIG homing endonuclease domain, one ORF encoding reverse transcriptase/maturase, and 3 ORFs with unknown functions.

**FIGURE 3:**
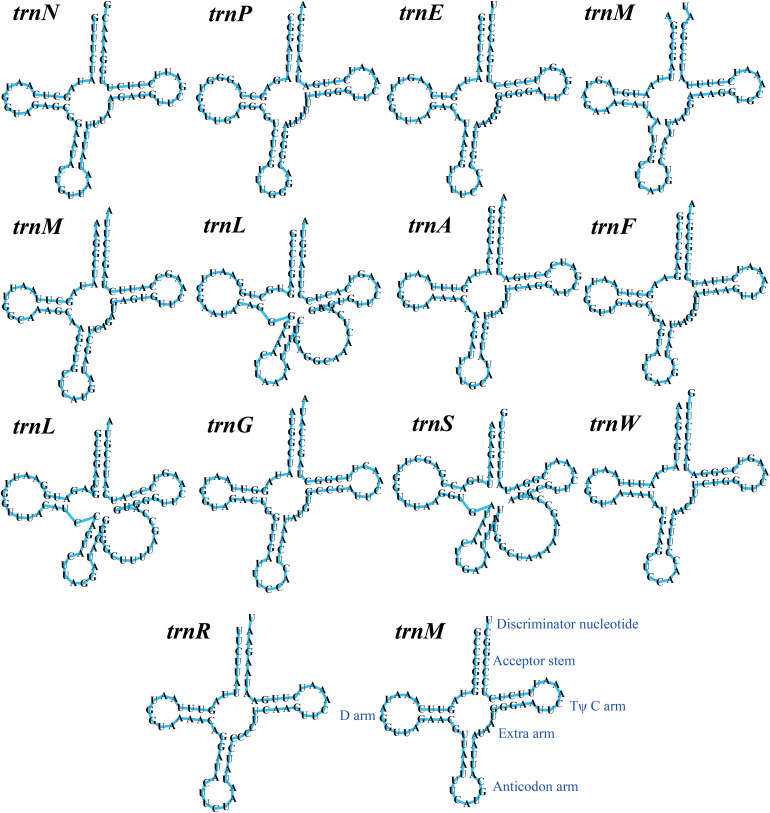
Putative secondary structures of the 14 tRNA genes identified in the mitochondrial genome of *Tuber calosporum*. All genes are shown in order of occurrence in the mitochondrial genome of *T. calosporum* starting from *trnN.*

The mitogenome of *T. calosporum* contained 2 rRNA genes, namely the small subunit ribosomal RNA (*rns*), and the large subunit ribosomal RNA (*rnl*) (Supplementary Table 1). Thirteen tRNA genes were detected in the *T. calosporum* mitogenome, which predicted structures resembled the classical clover leaf folding (Figure 3). The mitochondrial genome of *T. calosporum* lost *trnC, trnD, trnH, trnI, trnK, trnQ, trnT, trnV*, and *trnY*, which predicted function is the transport of cysteine, aspartate, histidine, isoleucine, lysine, glutamine, threonine, valine, and tyrosine, respectively. The mitogenome of *T. calosporum* contained two tRNAs with different anticodons coding for leucine and three tRNAs with the same anticodon coding for methionine. The length of individual tRNAs ranged from 71 to 88 bp.

**FIGURE 4:**
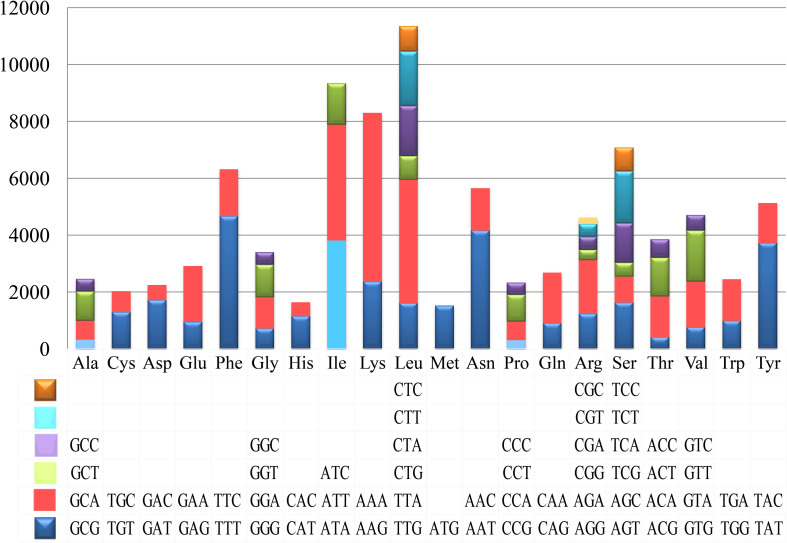
Codon usage in the mitochondrial genomes of *Tuber calosporum*. Frequency of codon usage is plotted on the *y*-axis.

Codon usage analysis indicated that the most frequently used codons in the mitogenome of *T. calosporum* were AAA (for lysine; Lys), TTT (for phenylalanine; Phe), TTA (for leucine; Leu), AAT (for asparagine; Asn), ATT (for isoleucine; Ile), and TAT (for Tyrosine; Tyr) (Figure 4 and Supplementary Table 2). The frequent use of A and T in codon contributed to the high AT content in the *T. calosporum* mitogenome (average: 70.08%).

### Repetitive Elements Analysis

We conducted BLASTN searches of the *T. calosporum* mitogenome against itself and identified 181 repetitive sequences in the *T. calosporum* mitogenome (Supplementary Table 3). The size of these repeats ranged from 35 to 999 bp, with pair-wise nucleotide similarities ranging from 74.68 to 100%. The longest repeats were detected in the third intron of *cox3* gene and also in the intergenic region of *trnM* and *cox1*. Repetitive sequences detected by BLASTN searches accounted for 12.83% of the *T. calosporum* mitogenome. Through Tandem Repeats Finder, we detected 15 tandem repeats (Supplementary Table 4), which accounted for 0.097% of the entire mitogenome. The longest tandem sequence (77 bp) was detected in the thirteenth intron of the *cox1* gene. Based on REPuter, a total of 50 forward repeats, accounting for 1.18% of the whole mitogenome, were detected (Supplementary Table 5).

We conducted BlastN searches of the *T. calosporum* mitogenome against its nuclear genome (Li H. et al., 2018), and identified 109 aligned fragments between the mitogenome and nuclear genome, with a total length of 32.91 kb (Supplementary Table 6). The length of these aligned fragments ranged from 102 to 1,360 bp, with sequence identities between 73.08 and 94.63%. The largest aligned fragment was found located in the sixth and seventh introns of *nad5 gene* and encompassed the seventh exon of *nad5* gene. The second largest aligned fragments were located in the third intron of *cox3* gene, and also in the intergenic region between *trnE* and *trnM*, with a length of 999 bp. The presence of large fragments aligned between the mitochondrial and nuclear genomes of the *T. calosporum* mitogenome indicated that genetic transfer between mitochondrial and nuclear genome has occurred in the evolution of *T. calosporum*.

### Comparative Mitogenomics

**FIGURE 5:**
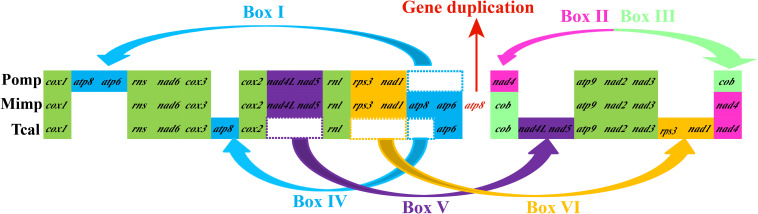
Mitochondrial gene arrangement analysis of 3 *Pezizales* mitogenomes. 15 core protein coding genes and 2 rRNA genes were included in the gene arrangement, starting from *cox1* gene.

Gene orders of the three Pezizales mitogenomes varied greatly between different species (Figure 5). We observed four gene relocations, two gene position exchanges, and one gene duplication event in the three Pezizales mitogenomes. Gene relocations involved box I, box IV, box V, and box VI, which harbored *atp6, atp8, nad4L, nad5, rps3*, and *nad1*. Gene position exchanges involved box II and box III, which harbored *nad4* and *cob* genes. The *M. importuna* mitogenome (Liu et al., 2019) had one duplication of *atp8* gene. Gene migration, transposition, and duplication observed in Pezizales mitogenomes indicated that frequent gene rearrangements occurred during the mitochondrial evolution of Pezizales species.

The mitogenome of *T. calosporum* was 5.57 and 50.32% larger than the two species from Pezizales, *M. importuna* (Liu et al., 2019) and *P. omphalodes* (Nieuwenhuis et al., 2019), respectively (Table 1). The GC content of the *T. calosporum* mitogenome was the lowest among Pezizales mitogenomes. Both the AT and GC skews were larger in *T. calosporum* mitogenome than in other two Pezizales mitogenomes, indicating high preferences for As and Gs in the *T. calosporum* mitogenome. The number of PCGs in *T. calosporum* was between *M. importuna* (the highest) and *P. omphalodes* (the lowest). However, the *T. calosporum* contained the greatest number of introns and intronic ORFs among the Pezizales mitogenomes detected. Two rRNA genes were detected in all the three Pezizales species. In addition, 14–31 tRNA genes were detected in the three Pezizales species. The mitogenome of *T. calosporum* had lost some of tRNA genes compared with the other two Pezizales mitogenomes.

### Phylogenetic Analysis

**FIGURE 6:**
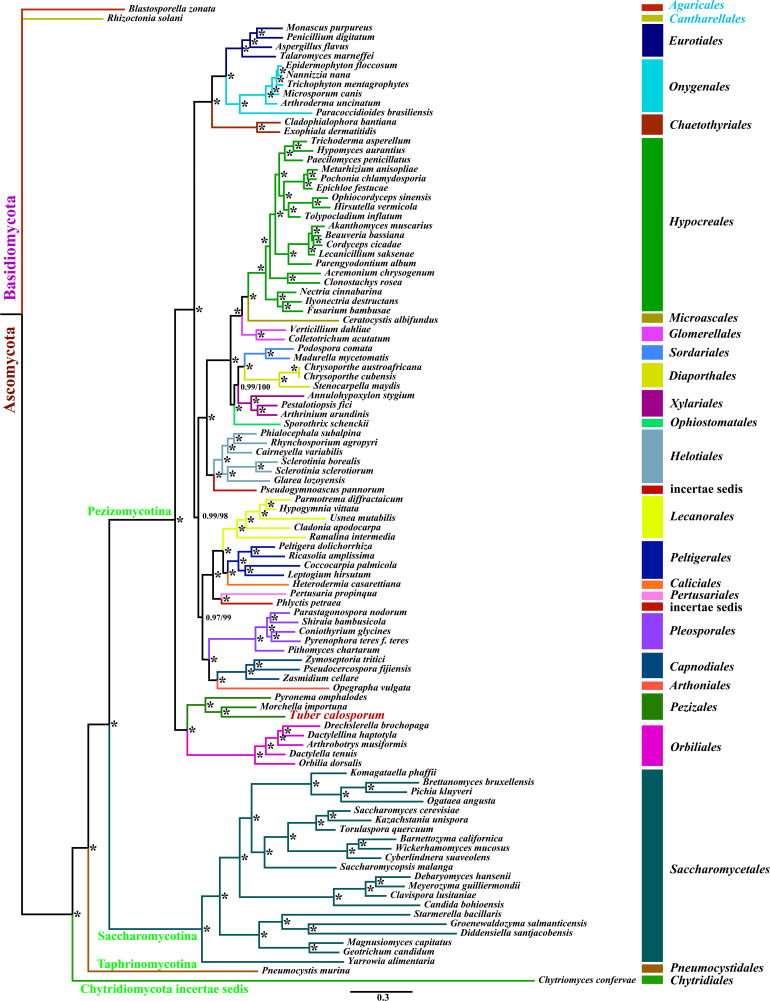
Molecular phylogeny of 104 fungal species based on Bayesian inference (BI) and Maximum Likelihood (ML) analyses of 15 protein coding genes and two rRNA genes. Support values are bayesian posterior probabilities (before slash) and bootstrap values (after slash). The asterisk indicates that the BPP and BS values are 1 and 100, respectively. Species and NCBI accession numbers for genomes used in the phylogenetic analysis are provided in Supplementary Table 6.

We obtained well-supported and identical tree topologies using maximum likelihood (ML) and bayesian inference (BI) methods based on the combined mitochondrial gene set (15 core PCGs + two rRNA genes) (Figure 6). All major clades within the trees had good support values (BPP ≥ 0.97; BS ≥ 98). Based on the phylogenetic analysis, the 101 Ascomycota species could be divided into 24 major clades, corresponding to the orders Diaporthales, Sordariales, Glomerellales, Chaetothyriales, Eurotiales, Onygenales, Xylariales, Microascales, Hypocreales, etc. The clade related to Pezizales was recovered as [*P. omphalodes*+ (*M. importuna* + *T. calosporu*)]. The result indicated that *T. calosporum* mitogenome had a close relationship with *M. importuna*. The phylogenetic analysis showed that mitochondrial genes were effective molecular markers for phylogenetic analysis of Ascomycota species.

## Discussion

The mitogenome was reported to have been derived from alphabacteria through endosymbiosis (Lang et al., 1999). In the long-term evolution, most of the ancient mitochondrial genes have been integrated into the nuclear genome, which is considered to have many advantages (Adams et al., 2002; Adams and Palmer, 2003). However, some genes also remain in the mitogenome for local control of oxidative phosphorylation and transcriptional regulation (Allen, 2015). Many physiological activities of eukaryotes need to be completed under the joint control of nuclear genome and mitogenome. The mitogenome size is more variable in fungi than in animals, which is believed to be closely related to the accumulation of repeat sequences, introns, plasmid related regions, and horizontal transferring genes (Himmelstrand et al., 2014; Mardanov et al., 2014). Up to now, the largest mitogenomes of Ascomycota and Basidiomycota are *M. importuna* (Liu et al., 2019) and *R. solani* (Losada et al., 2014), respectively. Accumulations of repeat sequences and introns contributed to the expansion of the two mitogenomes. In the present study, we obtained the mitogenome of *T. calosporum*. Compared with *M. importuna* and *R. solani*, the mitogenome of *T. calosporum* contained more introns and intronic ORFs. In addition, the intronic region accounted for 69.41% of the whole genome of *T. calosporum*, which was much higher than that of *M. importuna* and *R. solani*. The results indicated that intron gain was the primary factor contributing to the significant expansion of *T. calosporum* mitogenome, while the protein-coding region and intergenic region were reduced in *T. calosporum* mitogenome compared with the other three mitogenomes.

The GC content of mitogenomes varies between different species, which is thought to be affected by biases of reconstitution-related DNA repair, selection and mutation bias (Li et al., 2018c, 2019b). In the present study, we found the GC content of *T. calosporum* was significantly lower than the other two Pezizales mitogenomes reported. According to the second parity rule, each base in the complementary DNA strand exists at an approximately equal frequency if there is no mutation or selection bias (Chen et al., 2014). However, we found excesses of Gs and As (but not Cs and Ts) in the replication leading strands of Pezizales mitogenomes. Both the AT and GC skews were larger in *T. calosporum* mitogenome than in other 2 Pezizales mitogenomes, indicating unique evolutionary characteristics of the *T. calosporum* mitogenome.

Previous studies have shown that mitochondrial gene transfer to nuclear genome is a trend, which is considered having several advantages (Adams and Palmer, 2003). However, there were still a small number of genes that were transferred from nuclei to mitogenomes (Zhao et al., 2018). Natural gene transfer between nuclear and mitochondrial genomes showed great effects on species differentiation and functional evolution of mitogenomes (Adams et al., 2002). In the present study, large aligned fragments were observed between nuclear and mitochondrial genomes of *T. calosporum*, indicating gene fragments may have transferred between mitochondrial and nuclear genomes of *T. calosporum* in the process of evolution. Several non-conserved PCGs were also identified in the mitogenomes of *T. calosporum*, which encoded homing endonucleases, DNA polymerase, and other proteins with unknown function. These results suggest that there are still a number of undiscovered proteins in the *T. calosporum* mtiogenome that warrant future investigation. Homing endonucleases initiate transfer of introns, inteins, and themselves by generating strand breaks in cognate alleles that lack the intervening sequence, as well as in additional ectopic sites that broaden the range of intron and intein mobility (Stoddard, 2005). Interestingly, we found that the mitogenome of *T. calosporum* lost tRNA genes encoding for cysteine, aspartate, histidine, isoleucine, lysine, glutamine, threonine, valine, and tyrosine. Previous studies indicated that tRNA import and superwobble was the functional replacement for tRNA genes loss in mitogenomes (Rogalski et al., 2008; Salinas et al., 2008; Saunier et al., 2014; Warren and Sloan, 2020). Mitochondrial tRNA import has been experimentally documented in several organisms (Salinas et al., 2008). In contrast to mitochondrial protein import, tRNA import has a polyphyletic evolutionary origin (Schneider and Marechal-Drouard, 2000). Each organism recruits distinct housekeeping proteins to direct mitochondrial import (Salinas et al., 2008), but the overall process of mitochondrial protein import is conserved (Baker et al., 2007). ‘Superwobble’ in which a tRNA species with an unmodified U in the wobble position reads all four nucleotides in the third codon position, indicating that a reduced tRNA set could still suffice in some species (Rogalski et al., 2008). The functional replacement of tRNA gene loss and the effect to the synthesis of mitochondrial protein in *T. calosporum* needs to be further studied.

Mitochondrial gene arrangement can provide useful information for understanding the origin and evolution of species (Zheng et al., 2018; Li et al., 2020c), because mitochondrial genes of all species derived from a common ancestor (Lang et al., 1999). Mitochondrial gene rearrangements in animals have been studied extensively, and several models have been proposed to explain these rearrangement events (Boore, 1999; Perseke et al., 2008). Plant mitochondrial gene orders are highly variable owing to high recombination rates of their mitogenomes (Galtier, 2011; Liu et al., 2011). However, compared to other lineages, fungal mitochondrial arrangements have been less investigated, despite their importance in the global ecosystem (Hamari et al., 2001; Aguileta et al., 2014). In the present study, large-scale gene rearrangements were observed in the three Pezizales mitogenomes, which involved gene relocations, position exchanges, and duplication. Previous studies found that gene rearrangements in fungi were closely related to recombination and accumulation of repetitive sequences in fungal mitogenomes (Aguileta et al., 2014). Interestingly, we found the three Pezizales mitogenomes contained rich content of repeat sequences (over 12% of each mitogenomes). The accumulation of repeat sequences may lead to variable gene arrangements in the three Pezizales mitogenomes.

The *Tuber* genus is a group of important ectomycorrhizal fungi, which comprises numerous recognized species (Wan et al., 2016; Guevara-Guerrero et al., 2018; Polemis et al., 2019). Limited morphological features and overlapping of some features render it difficult to differentiate some *Tuber* species accurately. Reliable molecular markers are important tools for accurate identification and classification of *Tuber* species (Fan et al., 2016; Gryndler et al., 2017; Qiao et al., 2018), also for understanding origin of Pezizales species. Mitochondrial genes have been widely used in phylogeny and population genetic study of animals, plants, and some classes of fungi (Dai et al., 2018; Doyle et al., 2018; Li et al., 2019c; Wang et al., 2020). However, the number of known mitogenomes in Pezizales is very limited, which limits our understanding of the origin and evolution of Pezizales species. In this study, we obtained a well-supported phylogenetic tree based on combined mitochondrial gene set using two phylogenetic inference methods, which divided 101 Ascomycota species into 24 independent clades. The present study indicated that mitochondrial genes are reliable molecular markers to reconstruct phylogeny of Ascomycota.

## Data Availability Statement

The complete mitogenome of *T. calosporum* was deposited in the GenBank database under the accession number MT028548.

## Author Contributions

QL and XL conceived and designed the experiments. LL, XW, ZB, WT, XH, LY, and BZ analyzed the data. QL and XL wrote and reviewed the manuscript. All authors contributed to the article and approved the submitted version.

## Conflict of Interest

The authors declare that the research was conducted in the absence of any commercial or financial relationships that could be construed as a potential conflict of interest.

## Footnotes

1 http://www.dnastar.com/
